# Meharry Medical College Mobile Vaccination Program: Implications for Increasing COVID-19 Vaccine Uptake among Minority Communities in Middle Tennessee

**DOI:** 10.3390/vaccines10020211

**Published:** 2022-01-29

**Authors:** Donald J. Alcendor, Paul D. Juarez, Patricia Matthews-Juarez, Sheena Simon, Catherine Nash, Kirollos Lewis, Duane Smoot

**Affiliations:** 1 Center for AIDS Health Disparities Research, Department of Microbiology, Immunology and Physiology, School of Medicine, Meharry Medical College, 1005 Dr. D.B. Todd Jr. Blvd., Nashville, TN 37208-3599, USA; dalcendor@mmc.edu; 2 Department of Family & Community Medicine, Meharry Medical College, 1005 Dr. D.B. Todd Jr. Blvd., Nashville, TN 37208-3501, USA; pjuarez@mmc.edu (P.D.J.); pmatthews-juarez@mmc.edu (P.M.-J.); 3 Department of Internal Medicine, School of Medicine, Meharry Medical College, 1005 Dr. D.B. Todd Jr. Blvd., Nashville, TN 37208-3501, USA; ssimon@mmc.edu (S.S.); cnash@mmc.edu (C.N.); kilewis@mmc.edu (K.L.)

**Keywords:** mobile vaccinations, Tennessee, vaccines, COVID-19, disparities, minorities

## Abstract

To end or curtail the COVID-19 pandemic, it is essential to incorporate mobile vaccination programs into the national vaccination strategy. Mobile COVID-19 vaccination programs play an important role in providing comprehensive vaccination from federally qualified institutions to underserved communities facing a higher risk for COVID-19 acquisition. The Meharry Medical College COVID-19 mobile vaccine program (MMC-MVP) has provided lifesaving COVID-19 vaccines, free of charge, to communities throughout Middle Tennessee. Mobile deployment is vital for those forced to travel long distances to get vaccinated and who have limited access to medical providers or vaccine clinics, lack access to public transportation, or may be homebound. The MMC-MVP, established on 13 April 2021, via funding from the Bloomberg Foundation, is sourced with infectious disease experts, nurse practitioners, and community engagement personnel to provide COVID-19 vaccinations and information in a culturally competent manner to diverse communities in Middle Tennessee. To provide broader access to COVID-19 vaccinations and vaccine-related information, the MMC-MVP partnered with the Tennessee Community Engagement Alliance, Vanderbilt University School of Nursing COVID-19 vaccine strike teams, non-academic, community-based organizations, and faith-based organizations. During the September 2021 COVID-19 surge in Tennessee, the MMC-MVP provided nearly 5000 free COVID-19 vaccinations to targeted, underserved communities. The MMC-MVP has provided vaccine equity in communities with the highest risk for acquiring COVID-19 and with greatest need in this pandemic.

## 1. Introduction

Severe acute respiratory syndrome coronavirus-2 (SARS-CoV-2), the virus that causes COVID-19 disease and results in acute respiratory illness, is a newly emerged coronavirus that has reached pandemic levels as of March 2020 [1,2,3,4]. Infection with SARS-CoV-2 may produce asymptomatic and severe acute disease, which may be life-threatening, specifically in individuals with underlying medical conditions [5,6]. The recently developed and approved COVID-19 vaccines from Pfizer, Moderna, and Johnson & Johnson have proven to be safe and effective and have been shown to protect vaccinated individuals from severe disease, hospitalization, and death [7,8,9]. Therefore, vaccinating the unvaccinated is essential for mitigating these risks and preventing new infections [10,11]. The national strategy for the COVID-19 and pandemic preparedness program, established in January 2021 by the Biden administration, includes the development of a safe, effective, and comprehensive vaccination campaign as one of its seven goals [12]. In accordance with this comprehensive vaccination program, the United States (US) will spare no effort to ensure Americans achieve vaccinations quickly, effectively, and equitably [12]. The overall goal of the national vaccine campaign is to convert vaccines into vaccinations. Central to the success of this program is significantly improved vaccine allocation, distribution, administration, and tracking [12]. Vaccination programs that employ mobile vaccination units have played a key role in the distribution and administration of COVID-19 vaccines following emergency use authorization (EUA) of the current vaccines, as well as during the Pfizer vaccine’s expanded use for individuals five years and older [13]. The mobile vaccination programs have provided underserved communities with access to free COVID-19 vaccines and up-to-date information regarding vaccine safety and efficacy [14]. In an effort to “meet communities where they are”, mobile vaccine units and staff travel to coordinated vaccine events to provide COVID-19 vaccination to communities with limited access to medical providers or vaccine clinics and that lack access to public transportation—specifically those living in rural communities—to provide vaccines to individuals who may be homebound [15]. The impact of the COVID-19 pandemic has led to missed visits to health providers for scheduled vaccines and care, particularly among children in the midst of virtual school and sheltering in place recommendations. Mobile vaccine units aligned with health departments and federally qualified health centers (FQHCs) have been able to address parental concerns and state vaccine mandates for public schools by providing COVID-19 vaccines and CDC-recommended scheduled vaccines for children [16,17]. The poor uptake of the COVID-19 vaccine in the South has been problematic due to vaccine hesitancy and resistance, which is usually the result of misinformation and conspiracy theories targeting vaccine safety and efficacy [10]. In the state of Tennessee, low uptake of the COVID-19 vaccine is evident, particularly in rural and underserved urban communities [10]. These trends have been monitored by Meharry Medical College (MMC), from the beginning of the pandemic in Nashville, through its mobile COVID-19 testing campaign, which aims to provide free and unlimited COVID-19 testing to underserved minority communities at risk for COVID-19 acquisition. Following the Food and Drug Administration (FDA) EUA approval of the vaccines and allocation to FQHCs, it became clear that minority communities would need equitable access to free COVID-19 vaccines. With funding from the Bloomberg Foundation, the MMC COVID-19 mobile vaccine program (MMC-MVP) was established. Upon examining data from the mobile vaccination program at MMC, we have delivered free, life-saving COVID-19 vaccines to underserved communities, mainly African American and Hispanic/Latinx populations, in both urban and rural communities in Middle Tennessee. MMC-MVP provided vaccines on site at both residential and commercial locals and routinely collaborates with the Vanderbilt School of Nursing COVID-19 vaccination strike teams that go door to door providing vaccines to community members that are homebound and or disabled. The levels of vaccine hesitancy and vaccine resistance among community members was highly significant and largely centered around language/cultural barriers, distrust in the government and the medical establishment, concerns with adverse side effects of the vaccines, and confusion due to conspiracy theories about vaccine safety and efficacy. Here, we discuss the current impact of the MMC-MVP and its impact on the COVID-19 pandemic in underserved communities in Middle Tennessee and its future. We hypothesize that if we provide greater vaccine access, via the MMC-MVP, for underserved minority communities in Middle Tennessee to receive free and effective COVID-19 vaccines, that this would then reduce the spread of COVID-19 in these communities and subsequently reduce morbidity- and mortality-associated severe COVID-19 disease.

## 2. Materials and Methods

### 2.1. Meharry Medical College Mobile Vaccination Unit

On 13 April 2021, MMC received a generous gift from Bloomberg Philanthropies’ Greenwood Initiative, allowing for the expansion of mobile unit vaccine operations in and around Nashville and in satellite counties across Tennessee. The gift was part of a larger investment from Bloomberg Philanthropies to the nation’s four historically Black medical schools—MMC, Howard University College of Medicine, Morehouse School of Medicine, and Charles R. Drew University of Medicine and Science [18]—to expand their respective mobile unit COVID-19 vaccine operations in their local communities, as public health advocates have signaled the need for trusted vaccine administrators to combat lack of access and vaccine hesitancy within Black and medically underserved communities.

### 2.2. Candidates and Vaccinations

Potential candidates for vaccination arrived at prescheduled vaccine events and were asked by the MMC vaccine team whether they have been vaccinated for COVID-19; the team discussed available options if candidates wanted to receive the vaccine. Further discussion included candidate eligibility requirements, vaccination history, allergies, adverse reactions to prior vaccinations, and other information listed on the vaccine registration form. After the registration form had been completed and the information was logged into the database, the candidate received the first dose of the COVID-19 vaccine and was given a vaccination card containing critical information needed for the second dose.

### 2.3. Statistical Analysis

The data were collected and stored using Microsoft Excel files. Python software using Jupyter notebook was employed to clean, organize, group, and apply statistical methods to the data. Finally, Tableau was used to create visualizations representing grouped data.

## 3. Results

### 3.1. Meharry Medical College Mobile Vaccination Unit Outreach

The MMC-MVP was developed shortly after the FDA EUA of the Pfizer vaccine on 11 December 2020. There was an ongoing disparity in COVID-19 testing for underserved minority communities in Tennessee prior to the Pfizer vaccine’s emergency authorization. To address expected health disparities in COVID-19 vaccinations for minority communities in Middle Tennessee, MMC worked with the Bloomberg foundation to find a way to bring vaccines to communities at the greatest risk for severe COVID-19 disease. With aid of the foundation, the MMC-MVP has expanded to include additional communities in Middle Tennessee. Targeted populations for MMC-MVP included both rural and urban underserved minorities residing in public housing and communities with poor access to the health care infrastructure. We also targeted faith base communities, assisted living/elder care facilities, immigrant populations, and the homeless/unsheltered communities. We also included large corporate communities that experienced COVID-19 outbreaks and were interested in vaccinating their workforce.

**Figure 1:**
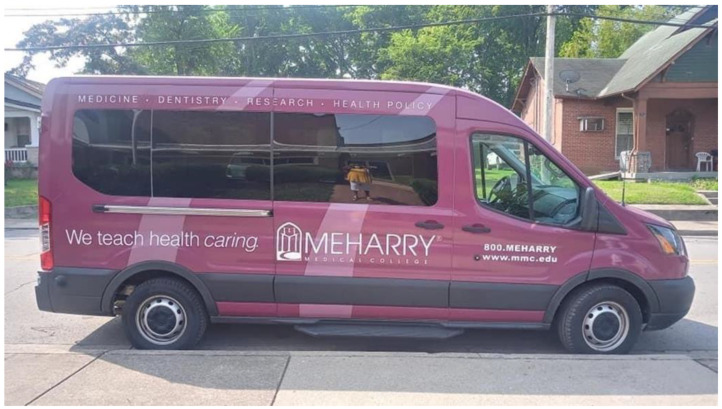
Meharry’s Mobile vaccine unit vehicle deployed to deliver COVID-19 vaccines throughout Middle Tennessee.

The mobile COVID-19 vaccination unit and staff are housed in the MMC School of Dentistry. Vaccine allocations for the mobile unit are provided by the State of Tennessee Department of Health. The mobile unit is deployed to travel to both urban and rural counties throughout Middle Tennessee to educate residents and administer FDA-approved COVID-19 vaccines from Pfizer, Moderna, and Johnson & Johnson to vaccine-eligible populations. The unit seats twelve staff members and specialty personnel and is equipped with special temperature-controlled coolers for temporary vaccine storage. The mobile unit is supported by highly trained MMC staff, including infectious disease experts, nurse practitioners, and community engagement personnel. The unit travels to prearranged vaccine event venues, such as federal housing facilities, churches, public and private businesses, Historically Black Colleges and Universities, elder care facilities, shelters, barbershops and salons, shopping malls, and park and recreation centers. Figure 1 is a picture of the van deployed in the MMC-MVP.

### 3.2. Mobile COVID 19 Vaccine Distributions in Middle Tennessee Reach Underserved Populations

**Figure 2:**
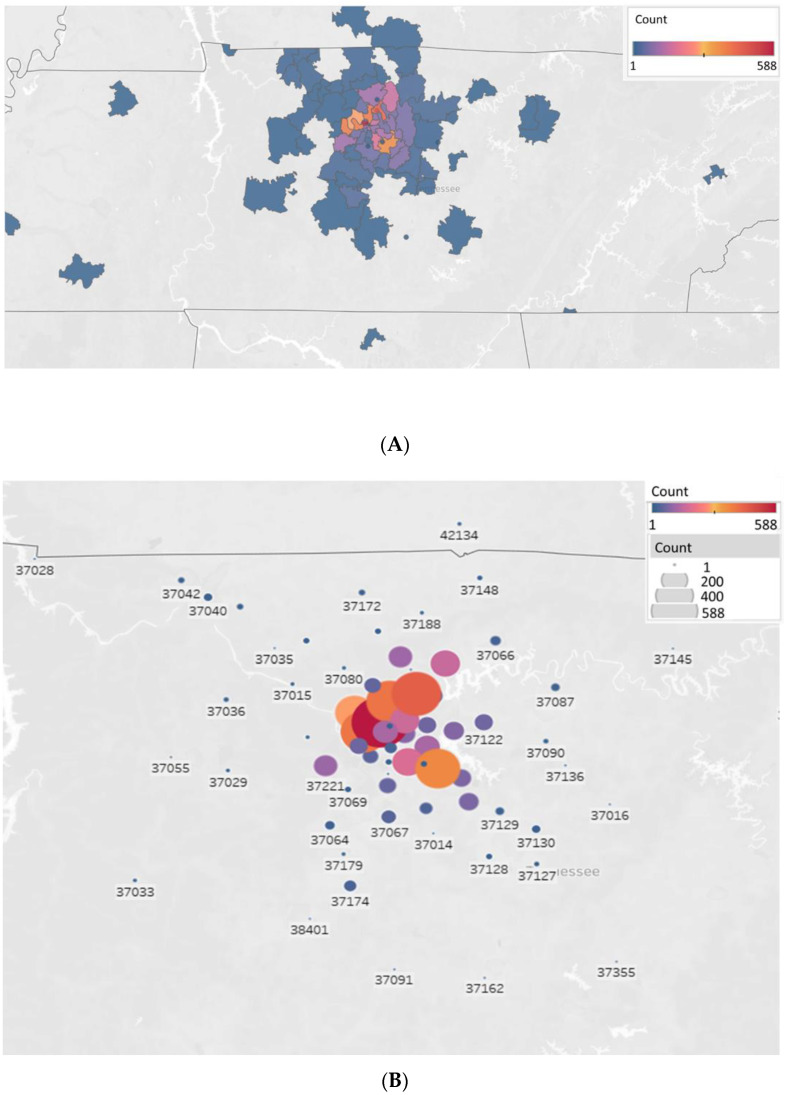
Regional COVID-19 vaccine distributions in Middle Tennessee using the MMC-MVP (**A**). Distribution of vaccines in Middle Tennessee by the Meharry Medical College-Mobile Vaccination Program by zip code; vaccination count represented by size and color (**B**).

**Figure 3:**
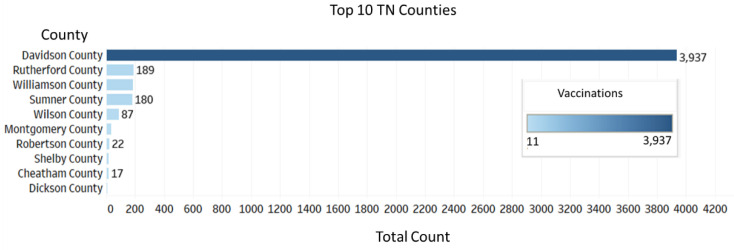
Number of vaccines distributed by the Meharry Medical College-Mobile Vaccination Program in the top 10 counties in Tennessee.

The long-term impact of COVID-19 on underserved communities in Tennessee has been devastating. Prior to COVID-19, underserved communities in Tennessee have struggled with economic hardships and health disparities. The COVID-19 pandemic exacerbated pre-existing inequities, highlighted by the social determinants of health (SDOH). Therefore, it became critical to expedite COVID-19 vaccine distributions to these communities, who are at higher risk for the more severe outcomes of COVID-19 disease. The MMC-MVP provided free COVID-19 vaccines and vaccine-related information to both urban and rural satellite counties in Middle Tennessee (Figure 2A). Vaccine events targeted underserved populations by zip codes based on vaccination rates and SDOH (Figure 2B). The zip codes include populations that are traditionally vaccine-hesitant or vaccine-resistant due to distrust of the government and/or medical establishment. The top ten counties served by the MMC-MVP included Davidson County, the second largest county in Tennessee, followed by Rutherford, Williamson, Sumner, Wilson, Montgomery, Robertson, Shelby, Cheatham, and Dickson Counties (Figure 3).

### 3.3. Meharry Medical College-Mobile Vaccination Program from March 2021 through COVID-19 Surge in September 2021

**Figure 4:**
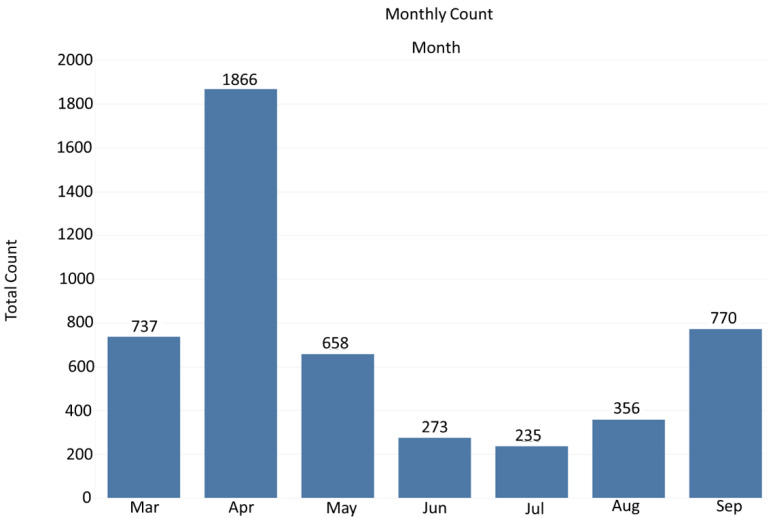
Total number of vaccines provided monthly by the Meharry Medical College-Mobile Vaccination Program from March 2021 to September 2021.

In March 2021, Tennessee was rebounding from the December 2020 surge in COVID-19 new infections and increased hospitalization rates [19,20]. On 30 August 2021, just prior to the September surge, the moving seven-day average was the highest of the pandemic, averaging 8442 new cases per day [21]. Data obtained from the MMC-MVP between March 2021 and September 2021 reveal that the vaccination team provided free COVID-19 vaccinations to 4895 participants during urban and rural community vaccine events (Figure 4). The highest number of vaccinations provided by the program during this time was achieved in April and September 2021 (Figure 4).

### 3.4. Meharry Medical College-Mobile Vaccination Program Reaches Targeted Populations Reluctant to Vaccinate

**Figure 5:**
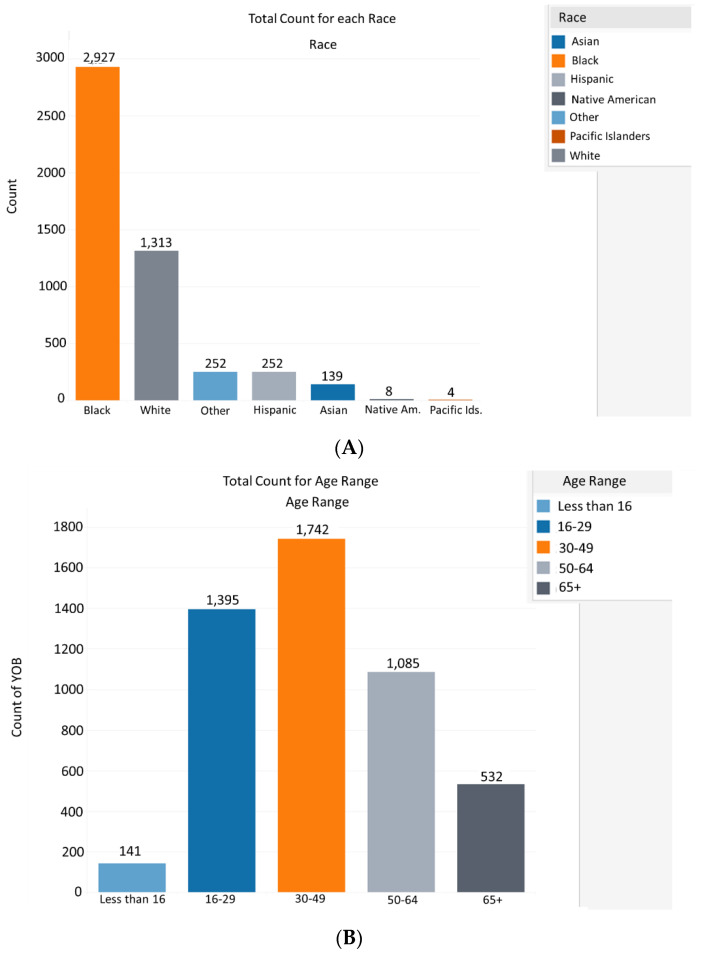
Total number of vaccinations provided by the Meharry Medical College-Mobile Vaccination Program by race (**A**) and by age (**B**).

To date, approximately 50% of Tennessee’s population has been fully vaccinated for COVID-19. However, young, underserved minority populations across the state have been reluctant to accept the COVID-19 vaccine. Blacks and rural non-Hispanic whites have the lowest fully vaccinated rates in Tennessee at 34% and 38%, respectively, and represent 17.1% and 78.4% of the state’s population, respectively [22]. In terms of age, the percentage of unvaccinated Tennesseans is highest among individuals aged from 16 to 40 [23]. As shown in Figure 5A, Black and non-Hispanic whites represent the majority of vaccinated candidates serviced by the MMC-MVP (Figure 5A). The MMC-MVP also provided COVID-19 vaccine access to the age demographic in Tennessee that is less likely to accept vaccination. As shown in Figure 5B, the MMC-MVP has provided more vaccinations for individual’s aged from 16 to 49 than any other age demographic.

## 4. Discussion

With nearly 5000 vaccinations administered to targeted communities at the greatest risk for the most severe symptoms associated with COVID-19 disease, we have impacted these communities in important ways to reduce severe disease, hospitalizations, and death due to COVID-19. The MMC-MVP has been significant in reducing community spread among the most vulnerable to COVID-19 infection.

Mobile COVID-19 vaccination programs are essential in providing free COVID-19 vaccine access and information to state, local, and tribal communities throughout the United States. Mobile vaccination programs are a cost-effective way of providing hard-to-reach and high-risk populations with COVID-19 vaccines. Some underserved community partners are often reluctant to accept the vaccines. There were cultural and language barriers that had to be addressed. The MMC-MVP provides on-site translators and bilingual medical staff. The staff also provides flyers, infographics, and Facebook Live sessions focused on vaccine safety and efficacy in different languages to accommodate community partners with English as a second language. Furthermore, the MMC-MVP partners with Hispanic/Latinx and immigrant community base organizations to support vaccine information in the appropriate cultural context.

With the continual evolution of new viral variants, breakthrough infections, unvaccinated populations, and long-standing global vaccine inequities, the end of the COVID-19 pandemic appears uncertain at this time [24,25,26,27,28]. SARS-CoV-2, the etiological agent for COVID-19, will likely become endemic in the US, resulting in continual spikes and possibly larger outbreaks, requiring the continued deployment of mobile vaccination program units. Vaccinating the unvaccinated in the US and around the world must be a high priority. This will require an extension of existing vaccine manufacturing and distribution into developing countries to expedite vaccine delivery. In resource-poor countries, where most of the population resides in rural communities requiring distant travel for health care access, mobile COVID-19 program units will play a significant role in bringing vaccines to these isolated, underserved communities. The need for COVID-19 testing and vaccinations is more pronounced in rural communities in Tennessee and throughout the South. Rural communities are overburdened with an extended travel time to health care services and lack access to public transportation. The lack of health care infrastructure and access to primary and acute care physicians in rural communities is a longstanding problem in Tennessee and throughout the South. Coupled with persistent vaccine hesitancy and resistance in these rural communities, COVID-19 vaccine uptake is likely to remain low.

In African American communities, it is essential to dispel misinformation associated with vaccine safety and efficacy. Vaccine information should be displayed and explained to communities and their questions answered via social media and town halls, as well as in non-traditional venues such as barbershops, salon, churches, and grocery markets. In addition, community liaisons could be employed to serve as trusted messengers to improve COVID-19 vaccine confidence. It is also essential to make vaccine access and COVID-19 testing available to all African American community members that reside in both urban and rural Tennessee, supporting the need for mobile testing and mobile vaccination programs.

To improve COVID-19 vaccine acceptance among rural non-Hispanic whites in Tennessee, we must mitigate vaccine hesitancy by developing messaging platforms targeting rural communities by enlisting the help of Country, Western, and NASCAR celebrities in Nashville, as well as by participating in community events such as the Harvest Festival and the Tennessee State Fair. Vaccine ambassador training would help form a team of ambassadors who can build trust and instill vaccine confidence within their respective rural community members. Vaccine ambassadors would also include parents, students, teachers, primary care physicians, and pastors. We must also debunk long-standing conspiracy theories involving the government and vaccine companies with evidence-based scientific information. We must develop pathways to connect rural communities in Tennessee to the existing COVID-19 prevention infrastructure. Finally, we must address the barriers that inhibit rural community access to COVID-19 testing and vaccines.

Mobile vaccine programs will play an important role in COVID-19 vaccinations and CDC-scheduled vaccinations of Afghan refugee resettlements in Tennessee and throughout the US. Tribal communities in the US have greatly benefited from mobile vaccination programs that also include expanded health care services such as dental screening, cholesterol and diabetes testing, blood-pressure screenings, hearing tests, and glaucoma screenings. Large corporations in the US have employed mobile vaccinations program units to vaccinate their workforce against COVID-19. Mobile vaccine units deployed to elder care facilities have been instrumental in mitigating COVID-19-associated morbidity and mortality in this vulnerable, at-risk population. Mobile vaccination program units are providing standard vaccinations for immigrant and migrant communities in Tennessee, especially for pediatric populations that have not been able to attend routine wellness visits. Mobile vaccinations programs can coordinate their events with community base organizations to increase their volunteer base and foster trust within communities to improve uptake of the COVID-19 vaccine. To reduce language and culture barriers, bilingual community partners can be integrated into the staffing to ensure all participants are engaged in a culturally competent manner. The social conservatism in Tennessee and the South will require mobile vaccine programs to partner with faith base communities to support the unique and necessary role that the COVID-19 vaccines will play in ending/curtailing this pandemic. Religious beliefs are deeply rooted for some individuals, and may preclude the use of vaccines to prevent infection, disease, and death. Therefore, vaccine program staff must engage pastors and other faith staff to establish a dialog with congregants to improve vaccine confidence. For vaccine distribution to these communities, mobile vaccination programs must partner with local health departments, public health clinics, pharmacies, and private-sector vaccinators to bring COVID-19 vaccinations to scale within these at-risk communities. The CDC and the Federal Emergency Management Agency (FEMA) have developed materials to help FQHC’s and other jurisdictions establish mobile vaccination sites and mobile vaccination units, and have expanded their use as COVID-19 vaccinations are needed. Mobile vaccination units and programs will become increasingly important in strategies to rapidly vaccinate more of the population, especially at a time when multiple boosters may be required to protect the population, due the continual emergence of SARS-CoV-2 variants that may evade the immune protection of existing vaccines. As stated in the Biden administration’s National Strategy for the COVID-19 Response and Pandemic Preparedness, the federal government will establish partnerships with state and local governments to create as many venues for vaccination as are needed, in communities and settings that people trust. These settings include mobile vaccine programs and mobile vaccination units. The national strategy focuses on hard-to-reach and high-risk populations, and meeting communities where they are to make vaccinations as accessible and equitable as possible. This can be achieved with the overall coordination of all national and international stakeholders, with a common goal of ending the COVID-19 pandemic on a global scale. This would include the global delivery of life-saving COVID-19 vaccines via mobile vaccination programs and mobile vaccination units to all global communities in need. This would require a meeting of nations and capital investments to include public and private partnerships in supporting resource-poor nations and ensuring equitable access and opportunities to benefit from the manufacturing and distribution of vaccines within each country. Training programs, designed to create a workforce for mobile vaccine programs and mobile vaccine units, are essential due to the current shortages of health care workers and overburdened medical care providers. We can expect that the role of mobile vaccine programs and mobile vaccine units will increase over time, as new infectious threats emerge.

## 5. Conclusions

At MMC, we have measured the success of our mobile COVID-19 vaccine program and would like to offer the MMC-MVP as a model for delivering COVID-19 vaccines to underserved communities throughout the South. In the future, the MMC-MVP wants to expand COVID-19 vaccination to additional rural counties across Tennessee, where vaccination rates are lowest. Expansion will not occur without challenges, due to the high level of vaccine hesitancy and resistance in these areas. We plan to partner with entities in rural counties, including primary care physicians, teachers, parents, and teenagers, to develop a cadre of vaccine ambassadors/trusted messengers among these individuals to promote COVID-19 uptake in their communities. At MMC, we will continue to provide COVID-19 vaccine testing and free COVID-19 vaccines to communities in need. Our program staff are committed to providing up-to-date COVID-19 information and services to underserved populations.

Taken together, the MMC-MVP has served both urban and rural communities to increase COVID-19 vaccination rates throughout Middle Tennessee.

## Data Availability

The study did not report any laboratory-based data.
